# Magnetoelastic Sensors-Based Pumpless Microfluidic Chip for Point-of-Care Coagulation Kinetics Monitoring

**DOI:** 10.3390/bios16080429

**Published:** 2026-08-06

**Authors:** Yao Lu, Weiguo Liang, Jun Qian, Junpo Li, Shengpeng Wu, Mao Xia, Haixuan Sun

**Affiliations:** 1 School of Biomedical Engineering (Suzhou), University of Science and Technology of China, Hefei 230026, China; 2 Suzhou Institute of Biomedical Engineering and Technology, Chinese Academy of Sciences, Suzhou 215163, China; 3 Department of Laboratory Medicine, Affiliated Hospital of Nantong University, Nantong 226001, China

**Keywords:** magnetoelastic sensor, coagulation kinetics, microfluidics, point-of-care, acute coagulopathy

## Abstract

Rapid hemostasis assessment is essential for managing acute coagulopathy, guiding anticoagulant therapy, and monitoring cardiac surgery patients. Although viscoelastic testing provides valuable guidance for early intervention, its widespread adoption is constrained by large blood usage (~2 mL), prolonged turnaround times (several hours), long assay durations (~25–40 min), and high costs. To overcome these limitations, we developed an innovative, all-in-one magnetoelastic (ME) sensing chip that enables systematic coagulation kinetics monitoring using only 46 μL of whole blood within 15 min. Featuring prepackaged lyophilized reagents and pumpless blood loading, this user-friendly chip is highly cost-effective for disposable use. The experimental results demonstrated good reproducibility for on-chip extrinsic coagulation activation, with clotting parameters maintaining coefficients of variation under 10%. Furthermore, clotting parameters derived from heparin monitoring results exhibited a strong linear correlation that compares favorably with the clinical standard (r = 0.986). Finally, sensitivity evaluation toward various blood components validated the sensor’s dual-mode characterization strategy for identifying coagulation factors and fibrin-related coagulopathies. The proposed ME sensing chip holds promise for bedside testing and flexible, on-demand coagulation monitoring across diverse clinical scenarios.

## 1. Introduction

Rapid and accurate coagulation monitoring is vital for the management of bleeding and thrombosis risks across a variety of clinical settings, including cardiovascular disease, stroke, hemophilia, surgery, trauma, sepsis, extracorporeal support, and antithrombotic therapies [1,2,3]. The early identification and correction of coagulation disorders are of significant importance in reducing transfusion requirements, improving patient outcomes, and reducing complications and mortality [2,4]. Standard clinical assays include plasma-based prothrombin time (PT) and activated partial thromboplastin time (aPTT) assays, whole blood-based activated clotting time (ACT) tests, viscoelasticity tests, and platelet function assays [4,5]. Among these, viscoelastic testing provides a global assessment of clot formation that is derived from coagulation factors, fibrinogen, and platelets, to guide perioperative bleeding management and targeted component therapy [6]. However, its widespread adoption is hindered by prolonged turnaround times, professional operations, and excessive blood consumption, which impede its application at point-of-care (POC) and emergency settings. Crucially, highly frequent and large-volume blood withdrawal for clinical assays can cause diagnostic blood loss, leading to serious iatrogenic anemia and adverse events [7,8].

Rapid, user-friendly near-patient hemostasis monitoring is critical for timely clinical decision-making in patients with acute coagulopathy, those undergoing anticoagulation therapy, or in the ICU [2,9,10]. Consequently, various near-patient hemostasis analyzers have been developed [11,12]. For instance, the TEG^®^ 6 s system (Haemonestics, Boston, MA, USA) employs a resonance method to measure clot viscoelasticity in real time at the bedside [13,14]. It pipettes the blood sample into a microfluidic cartridge with four channels where dried reagents are reconstituted via valves and bellows. Despite this ingenious design, it still requires approximately 0.4 mL of blood and involves higher per-test costs [13]. Conversely, while portable endpoint method-based coagulometers like i-STAT (Abbott, Princeton, NJ, USA) [15] and CoaguChek (Roche Diagnostics, Mannheim, Switzerland) [16] offer simple PT/INR or ACT values by fingerstick sampling to help patient self-monitoring, they cannot capture coagulation kinetics, such as clot grow rate and clot strength.

To bridge this gap, acoustic technologies have been widely explored for clot viscoelasticity measurements due to their rapid response and high sensitivity. Micro-electromechanical system (MEMS) devices, such as quartz crystal microbalance (QCM) [17,18], surface acoustic wave (SAW) [19], microcantilever [20,21,22], membrane capacitance [23], and film bulk acoustic resonators (FBAR) [24,25], have driven significant advancements in coagulation sensing. Acoustic waves propagating into blood alter the sensor’s electrical properties (resonance frequency and amplitude) as the clot evolves [26]. During blood coagulation, the polymerization of fibrin and the aggregation of platelets transition the rheological properties of blood from a non-Newtonian fluid to a Maxwellian fluid, reflecting dynamic changes in viscoelasticity [27,28]. Consequently, the clot formation can be quantitatively characterized by the electrical parameters of acoustic sensors. Nevertheless, for acoustic sensors operating at gigahertz frequencies, frequency measurement is tricky on a portable device [25,29]. More importantly, high fabrication costs often make them unsuitable for one-time-use. Although a disposable Love-mode SAW sensor was designed through a plug-and-paly-type connection for hemostasis assessment [19], it still requires wire-bonding and discards the attached heater module after a single use, wasting valuable resources. In addition, ultrasonic-based techniques offer significant advantages for coagulation monitoring due to their non-invasive and contactless detection. Deng et al. developed a resonant acoustic rheometry (RAR) approach to quantify the coagulation kinetic of plasma [30], and further achieved high-throughput screening by transducer array [31,32]. However, this method typically requires large blood volumes to avoid boundary interference caused by beam divergence and near-field dead zones. Additionally, its reliance on high-voltage driving circuits and acoustic coupling materials severely hinders equipment miniaturization, making it more suitable for non-invasive in vivo monitoring, or contactless high-throughput analysis in centralized laboratories [33,34].

To address these challenges, we utilized magnetoelastic (ME) sensors for rapid and easy coagulation measurement. Due to the magnetostrictive effect, ME sensors produce mechanical vibration (acoustic waves) with a frequency of tens to hundreds of kilohertz under an alternating magnetic field, making them highly attractive for biomedical applications [35,36,37]. Given their low cost, ease of fabrication and wireless detection [35], ME sensors are an ideal alternative for the development of rapid hemostasis assessment devices. Although a resonant amplitude-based ME biosensing method and a compact device were established for coagulation monitoring, the study lacked an investigation approach with systematic sensitivity for blood components, and the device integration remained inadequate for practical POC applications [38]. Here, we present a disposable, all-in-one ME sensor-based coagulation measurement chip characterized by microliter-scale blood consumption, easy operation, and a rapid sample-to-result workflow. Our systematic studies highlight an advancement in the dual-mode characterization of coagulation function compared to previous ME sensor-based research. By incorporating prepackaged lyophilized reagents, we successfully demonstrated the on-chip monitoring of both intrinsic and extrinsic coagulation pathways using clinical samples. Crucially, real-time viscoelastic testing demonstrated good reproducibility, and the heparin monitoring results exhibited strong agreement with the clinical standard, exhibiting its practical clinical utility. Furthermore, the selective and sensitive responses of the ME sensor to individual blood components provided deeper insights into the hemostatic disorders and microscopic physical properties of clots. This work underscores the potential use of ME sensors in POC devices, offering a promising solution for decentralized testing and real-time bleeding management at bedside or in emergency settings.

## 2. Materials and Methods

### 2.1. Reagents and Equipment

Amorphous alloy ribbon 1k101s (Fe_80_Si_9_B_11_) was purchased from Advanced Technology & Material Company Ltd. (Beijing, China). The poly (acrylic acid)-polyethylene glycol (PAA-PEG) mixed solution for the hydrophilic treatment of the materials’ surface was obtained from Dingxu Micro-control Technology Co., Ltd. (Suzhou, China). Kaolin, calcium chloride (CaCl_2_) and heparin sodium (≥180 U/mg) were purchased from Sigma-Aldrich (Shanghai, China). The recombinant human tissue factor (TF) was purchased from Beijing Bo’erxi Technology CO., Ltd. (Bersee, Beijing, China). Bovine fibrinogen and thrombin were purchased from Yeasen Biotechnology Co., Ltd. (Shanghai, China). The ACT Plus^®^ analyzer (Medtronic Inc., Minneapolis, MN, USA) was used to conduct comparative experiments. The activator (mainly containing 12% kaolin and 0.05 M CaCl_2_) from a high-range (HR) ACT cartridge supplied with the ACT Plus^®^ analyzer was used for heparin anticoagulation assessment.

### 2.2. Measurement Principle

**Figure 1:**
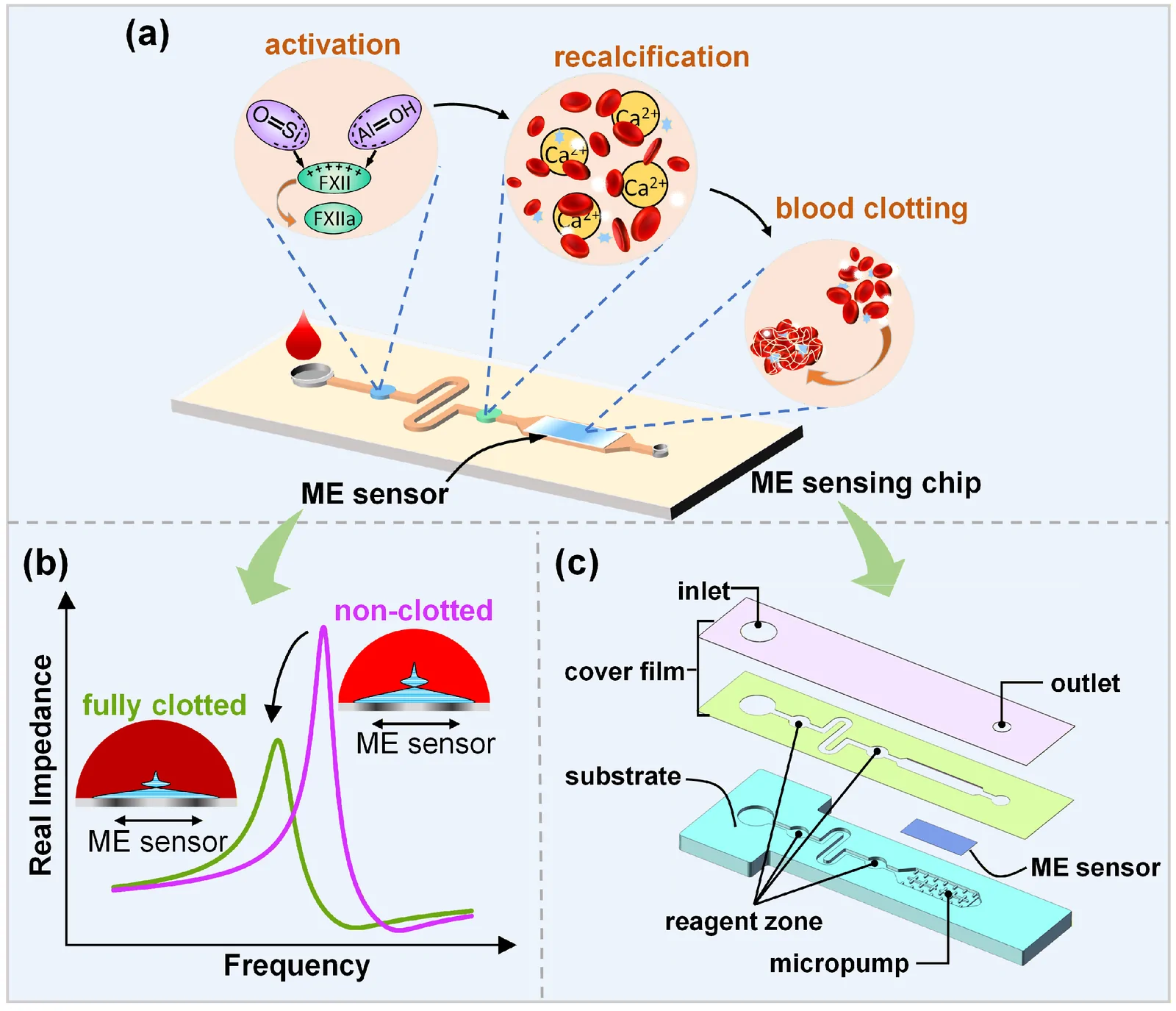
Schematic diagram of coagulation assay on magnetoelastic (ME) sensing chip. (**a**) Conceptual illustration of the sensing chip, depicting whole blood (WB) sample loading, capillary-driven transport for sequential activation and recalcification, and subsequent clot formation within the ME sensor-integrated region. "+" and "-" denote the positive and negative electric charges. (**b**) Working principle of the ME sensor, illustrating the changes in electrical real impedance and resonant frequency induced by the damping effect during clot formation (from non-clotted to fully clotted status). (**c**) Exploded view of the ME sensing chip, consisting of the cover film, the ME sensor, and the polystyrene (PS) substrate embedded with the reagent zones and capillary micropump.

To satisfy the needs for low blood consumption and operational simplicity across diverse clinical scenarios, we combined the ME sensor with capillary microfluidics to develop a pumpless microfluidic chip. Depending on the purpose of the coagulation assay, different activators were prepackaged within the microchannel. Kaolin triggers the intrinsic coagulation pathway, whereas a combination of kaolin and TF activates both the intrinsic and extrinsic pathways. As illustrated in Figure 1a, the capillary force drives the loaded whole blood (WB) sample to sequentially contact the prepackaged activators and calcium ions (Ca^2+^), thereby triggering the coagulation cascade reactions and ultimately generating thrombin and fibrin. The clotting measurement starts immediately once the blood fully submerges the ME sensor.

The high-frequency vibration produced by the ME sensor upon the excitation of the magnetic field is highly sensitive to the viscoelasticity of surface loadings. A theoretical model predicting the electrical behaviors of the ME sensor in response to different surface loadings has been established in our previous work [39]. Briefly, when the sensor is in contact with a viscous liquid, it experiences damping forces that cause a portion of its vibration energy to dissipate at the solid–liquid interface. This effect is electrically manifested as a downshift in resonant frequency and a reduction in impedance amplitude (Figure 1b). Therefore, as the coagulation cascade progresses, the evolving viscoelasticity of the blood clot can be detected by the ME sensor in real time. In this study, a clotting profile, established by tracking the peak amplitudes of the impedance–frequency curves of the ME sensor in real time, was used to characterize the entire coagulation process.

### 2.3. Design of ME Sensing Chip

The hydrophilic surface properties and microstructures of the device enable spontaneous fluid flow driven by the capillary effect. Based on this, we constructed the pumpless microfluidic chip, as depicted in Figure 1c. The chip comprises a substrate, an ME sensor, and a sealing cover film. Two reagent zones are set within the serpentine microchannel (1 mm wide, 0.4 mm high) on the substrate, where the reagents are prepackaged before assembly. The serpentine microchannel serves a dual purpose; its geometry introduces localized disturbances to facilitate the initial passive mixing between activators and blood, while increasing hydrodynamic resistance to dampen the flow velocity and lower the shear rate in the downstream detection region. Benefiting from its ultra-thin profile (17 μm thickness), the ME sensor can be seamlessly integrated within the microchannels without disrupting blood loading. Furthermore, a capillary pump with an array of micropillars (0.2 mm high) is set on the substrate to drive the blood sample over and around the sensor. The ME sensor is freely placed on the micropump structure to prevent vibration damping. To facilitate straightforward assembly, the cover film with inlet, reagents zones, microchannel, and outlet are bonded to the substrate using double-sided adhesive (DSA) tape.

### 2.4. Fabrication of ME Sensing Chip

**Figure 2:**
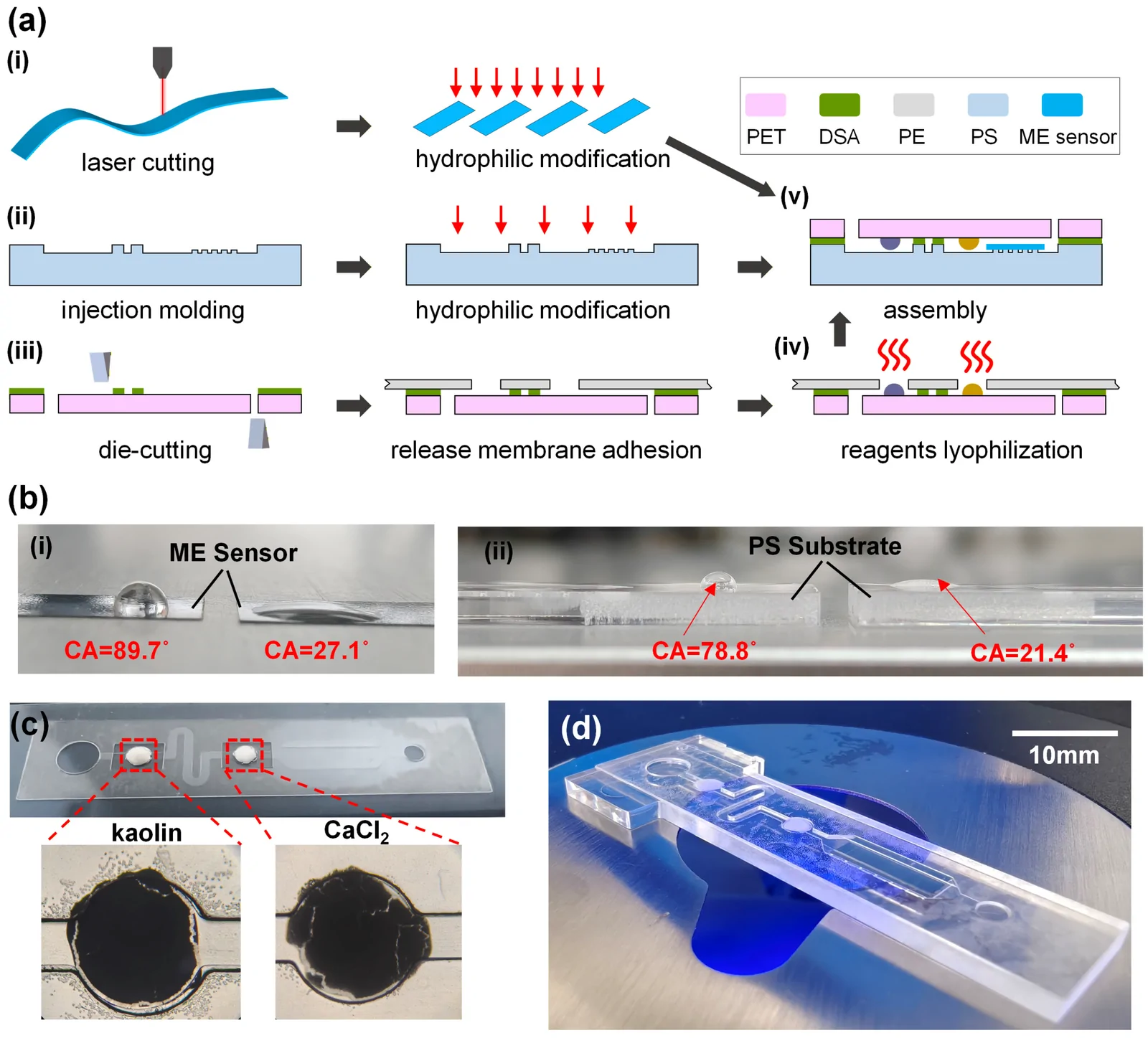
Fabrication of ME sensing chip. (**a**) Schematic fabrication process of the chip: (i) ME sensor dicing and modification, (ii) substrate injection molding and modification, (iii) cover film patterning, (iv) reagents lyophilization, and (v) assembly. (**b**) Hydrophilic modification profiles of the (i) ME sensor and (ii) PS substrate, with corresponding water contact angles (CA) annotated below. (**c**) Photograph of the prepared cover film alongside microscopic views of the lyophilized reagents (kaolin and CaCl_2_) on the reagent zones. (**d**) Photograph of the completely assembled ME sensing chip containing the prepackaged reagents.

The fabrication process of the ME sensing chip is schematically presented in Figure 2a. As shown in Figure 2a(i), the ME sensors were diced into rectangular strips (10 mm long, 4 mm wide) by laser cutting (Jiangyin Deli Laser Solutions Co., Ltd., Wuxi, China) from a 17 μm-thick amorphous alloy ribbon (0.18–0.36 USD/m). The diced sensors were ultrasonically cleaned in acetone for 10 min, sequentially rinsed in isopropanol and DI water for 5 min each, and then dried under a nitrogen stream. Afterward, the PAA-PEG solution was uniformly spray-coated onto the sensor surface to render it hydrophilic. Experimental validation confirmed a water contact angle (CA) below 40° (Figure 2b(i)), ensuring successful capillary-driven blood flow across the sensor surface within the chip.

To enhance the accessibility of the chip for practical applications, cost-effective batch manufacturing approaches were adopted. As shown in Figure 2a(ii), the substrates were fabricated by the injection molding of polystyrene (PS) (Suzhou Singmed Medical Device Science and Technology Ltd., Suzhou, China). The cover films were prepared from DSA and polyethylene terephthalate (PET) using a precision die-cutting technique (Suzhou Zhuanxin Electronic Materials Co., Ltd., Suzhou, China) to define the inlet, microchannel geometry, reagent zones and outlet (Figure 2a(iii)). A microchannel with a height of 450 μm was formed upon laminating the cover film (PET-DSA) onto the PS substrate. Besides this, the substrate surface was treated using the same method described above to render the surface hydrophilic (Figure 2b(ii)), thereby providing appropriate capillary force within the microchannel to drive the blood.

Prior to chip assembly, precise volumes of kaolin (0.2%), CaCl_2_ (0.3 M), and stabilizing agents were dispensed to their corresponding reagent zones on the cover film. The concentrations and compositions of on-chip lyophilized reagents were screened according to the state of the blood clot upon reaction completion, as inappropriate recipes could lead to uncertain dissolution and insufficient coagulation activation. For both the intrinsic and extrinsic pathways, TF (0.05%) was added into the kaolin reagent zone. The cover films containing the liquid reagents were then transferred to a freeze dryer (Laboratory Freeze Dryer, Tofflon, Shanghai, China) to undergo lyophilization (Figure 2a(iv)). A prepared cover film with successfully lyophilized reagents is shown in Figure 2c. Finally, the prepared ME sensor was placed onto the micropump of the substrate, and the cover film was laminated over them (Figure 2a(v),d). This configuration ensures that the ME sensor remains free to vibrate, and the lyophilized reagents can be stored stably under sealed, dry conditions. Based on a rough estimation of the materials and processing, the manufacturing cost of a single chip is approximately 1.66 USD (see Table S1 for details).

### 2.5. Measurement Setup

The experimental setup used for clotting measurements is illustrated in the Supplementary Information (SI) and Figure S1. Briefly, a compact, custom-designed detection module was developed to match the sensing chip. To characterize the coagulation process, the peak amplitudes of the impedance spectra of the ME sensor were measured in real time using a high-precision impedance analyzer (Micro Test 6632, SAIMR, Shanghai, China). All testing procedures were conducted under controlled room-temperature conditions.

### 2.6. Sample Preparation

Citrated (3.2%) human WB samples were obtained from donors at the Affiliated Hospital of Nantong University. A quality control (QC) material developed by our laboratory was used for the performance evaluation of the sensing chip. This matrix was derived from citrated (3.2%) porcine WB and comprised 40% (*v*/*v*) red blood cells (RBCs), with the remaining consisting of plasma, buffers, and stabilizing agents. This mixture was then prepared via freeze-drying. It should be reconstituted with deionized water before use. This reconstituted matrix is referred to as plasma-RBC QC (P-RBC QC) material throughout this article. In addition, a plasma-only QC material (referred to as plasma QC) was developed using the same method. To isolate specific blood components, fresh citrated human WB samples were centrifuged at 1000× *g* for 10 min. The upper plasma and buffy coat were transferred to a new tube and centrifuged at 1500× *g* for 10 min. The resulting supernatant was collected as platelet-poor plasma (PPP), while the platelet pellet was resuspended in a small volume of plasma to obtain platelet-rich plasma (PRP). Concurrently, the erythrocyte pellet from the initial centrifugation step was washed with saline and re-centrifuged to yield purified RBCs.

### 2.7. Heparin Anticoagulation Assessment

Heparinized blood samples with concentrations of 0, 0.5, 1, and 2 U/mL were prepared by adding heparin sodium solutions (0, 12.5, 25, 50 U/mL) to citrated WB at a ratio of 1:24. The resulting heparinized blood was incubated at room temperature for 5–10 min and then mixed with activators obtained from commercial HR-ACT cartridges at an 8:1 ratio in a centrifuge tube. Then, 46 μL of the mixture was pipetted onto a sensing chip, which was immediately placed into the detection module (described in Section 2.5) for coagulation monitoring. To ensure homogenization and assay reproducibility, manual pipetting in a centrifuge tube was intentionally preferred over on-chip passive mixing, given the high sensitivity of heparinized blood to mixing conditions. To validate the proposed method against the clinical standard, comparative testing was performed using an ACT Plus^®^ analyzer based on an endpoint detection method. For each ACT assay, 400 μL of heparinized blood was introduced into its cartridge maintained at 37 °C. The blood and liquid reagents were mixed by a reciprocating mechanical assembly, and the ACT value was obtained upon assay completion. To prevent sample degradation, freshly prepared heparinized blood was used immediately for every individual measurement.

## 3. Results and Discussion

### 3.1. Fluid Dynamic Analysis and Blood Flow Evaluation

**Figure 3:**
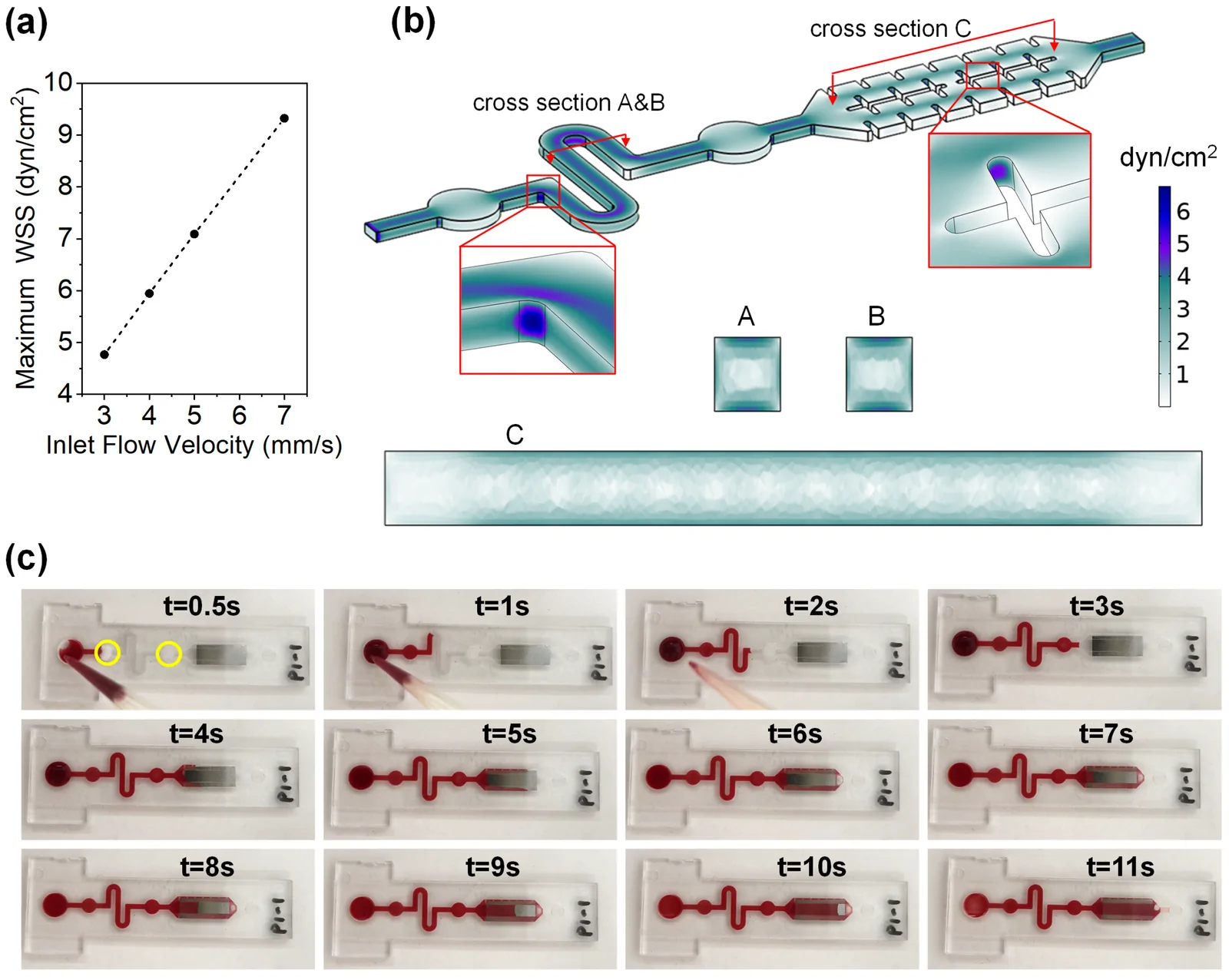
Modeling and experimental evaluation of blood flow within the ME sensing chip. (**a**) Simulated maximum wall shear stress (WSS) across the entire fluidic domain under various inlet flow velocities (3–7 mm/s). (**b**) Three-dimensional shear stress distribution at an inlet velocity of 5 mm/s, with localized magnification highlighting the maximum WSS (~6.7 dyn/cm^2^) at the microchannel corner. Labels A, B and C denote the internal cross sections within the microchannel and micropump regions. (**c**) Experimental snapshots of WB sample flowing within the sensing chip (marked circles indicate the prepackaged lyophilized reagents).

Platelets are highly susceptible to activation when exposed to transient, nonphysiological high shear stress [40]. This activation not only enhances their aggregation capacity and thus affects clot strength, but also accelerates the coagulation cascade, leading to a hypercoagulable state. Typically, physiological shear stresses in human arteries range from 10 to 40 dyn/cm^2^, corresponding to shear rates of 300 to 1200 s^−1^ [41,42]. To calculate the hydrodynamic characteristics of the designed chip, a three-dimensional laminar model was established (see SI for model details). As shown in Figure 3a, the calculated maximum wall shear stresses (WSS) within the microchannel remain below 10 dyn/cm^2^ under different inlet flow velocities (3–7 mm/s), indicating a hemodynamically safe microenvironment. Practically, the serpentine microchannel is 30.1 mm in length, with a blood flow time of 2.7–3.3 s, yielding flow velocities of 9.1–11.2 mm/s (4.1–5.0 μL/s) within the channel. When the inlet velocity was set to 5 mm/s in the model, the computed flow velocity in the serpentine channel reached 9.5 mm/s (Figure S2a), which matched well with experimental observations. Additionally, the shear stresses across the entire fluidic domain at an inlet velocity of 5 mm/s are presented in Figure 3b, where the maximum WSS (~6.7 dyn/cm^2^) appears at the microchannel corner. Notably, the internal shear stresses at cross-sections A, B, and C in both the microchannel and micropump remain sufficiently low. Further simulation results regarding flow velocities and shear rates are provided in Figure S2 of the SI. These results demonstrate that the capillary-driven flow within our chip does not induce platelet activation, thereby ensuring a biocompatible fluidic microenvironment for reliable coagulation analysis. Figure 3c displays sequential snapshots of a 46 μL WB sample introduced into the chip with prepackaged reagents (a video is provided in the SI). The blood flowed through the entire chip within approximately 11 s, with a flow velocity of ~10 mm/s within the serpentine microchannel. At this flow velocity, the shear stress exerted on the blood closely matches the simulated results in Figure 3b, thereby preventing shear-induced platelet activation.

To evaluate the dissolution and convective mixing of the prepackaged lyophilized reagents under such flow conditions, a color indicator was used in the reagents for visualization (Figure S3). A 40 wt% glycerol aqueous solution (viscosity: ~3.65 mPa·s), simulating the WB sample, was introduced to the chip. The captured snapshots reveal that the porous, red-dyed lyophilized reagents dissolve rapidly in the glycerol–water solution and diffuse across most of the ME sensor detection zone. This level of mass transport is sufficient to trigger the coagulation cascade, as the clotting process initiates locally and propagates throughout the detection region. Subsequent performance validation confirmed that the on-chip lyophilized reagents dissolved in the flowing blood maintain good consistency across multiple coagulation measurements. This design represents a balanced trade-off between user-friendly, capillary-forced flow and efficient passive mixing within such a portable coagulation monitoring device.

### 3.2. Performance Validation of the Chip

**Figure 4:**
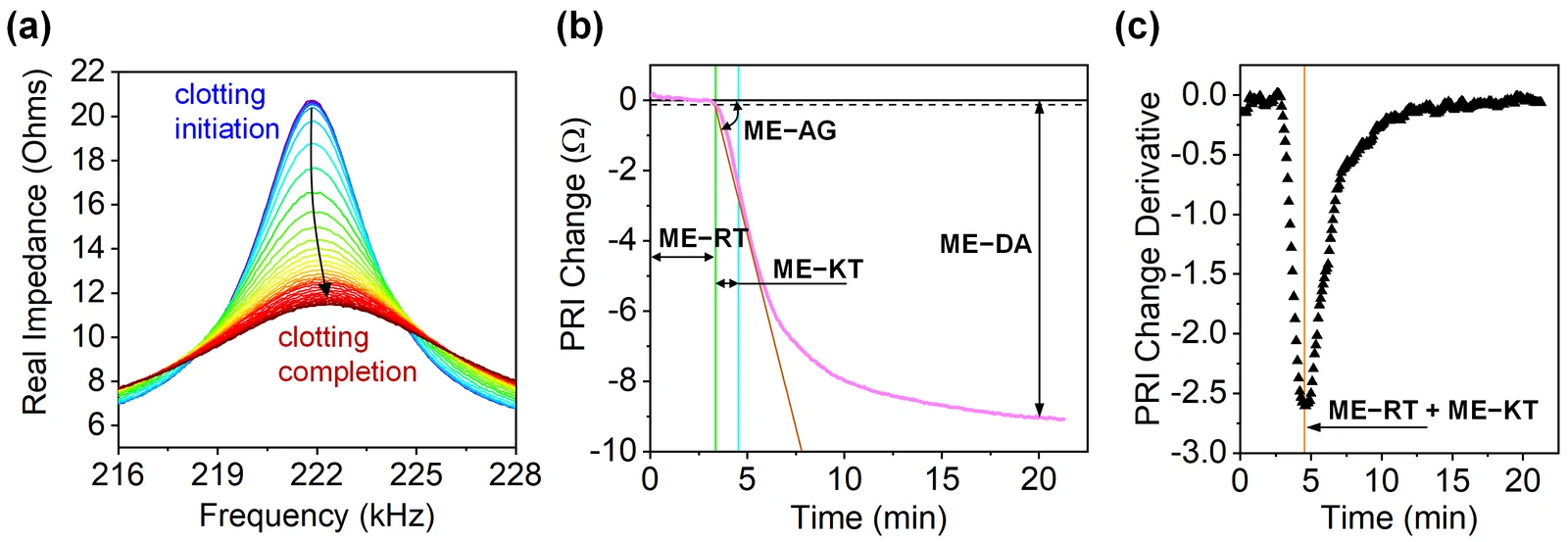
Coagulation characterization of ME sensing chip with prepackaged activators. (**a**) Real impedance–frequency curves recorded during the clotting of plasma-red blood cell quality control (P-RBC QC) samples (sampling interval: ~5 s). (**b**) Clotting profile constructed by tracking the change of peak real impedance (PRI) over time. Four clotting parameters were defined to quantitatively characterize the coagulation process: ME-reaction time (ME-RT, min), ME-kinetics time (ME-KT, min), ME-angle (ME-AG, °), and ME-delta amplitude (ME-DA, Ω). (**c**) The first derivative of the clotting profile, with its minimum point defining the time of maximum clotting velocity (ME-RT + ME-KT).

By loading a droplet of the P-RBC QC sample onto the sensing chip prepackaged with activators (kaolin and CaCl_2_), coagulation was initiated, and the real impedance–frequency curves of the ME sensor during clot formation were measured (Figure 4a). Owing to the viscous damping effect exerted by the developing blood clot, the peak real impedance (PRI) progressively decreased until the coagulation was complete. This time-dependent change, reflecting clot evolution, was recorded and further processed by subtracting a baseline (mean PRI in the un-clotted state) from each PRI, thereby yielding the relative change in PRI over time (Figure 4b, pink trace). We defined this curve as the clotting profile. To quantitatively evaluate the entire clotting process, we further defined four clotting parameters, as labeled in Figure 4b. (1) ME-reaction time (ME-RT): the time elapsed from the baseline to a predefined threshold of amplitude change (−0.12 Ω), denoting the onset of clot initiation. (2) ME-kinetics time (ME-KT): the time interval from ME-RT to the point corresponding to the minimum of the first derivative of the clotting profile (Figure 4c), representing the maximum clot growth rate. (3) ME-angle (ME-AG): the maximum angle formed between the baseline and the line connecting the reaction onset and a subsequent point on the curve, reflecting the global clot growth dynamics. (4) ME-delta amplitude (ME-DA): the maximum net change in PRI during the coagulation process, quantifying the clotting strength. Establishing clinical reference ranges for these parameters could facilitate the diagnostic classification of hemostatic disorders. This multi-parameter approach is analogous to thromboelastography (TEG), where multi-parameter integration is necessary to accurately identify hypercoagulable or hypocoagulable states. Additionally, the resonant frequency in Figure 4a shows a small upward trend and is depicted in Figure S4, which will be discussed in a subsequent section.

**Figure 5:**
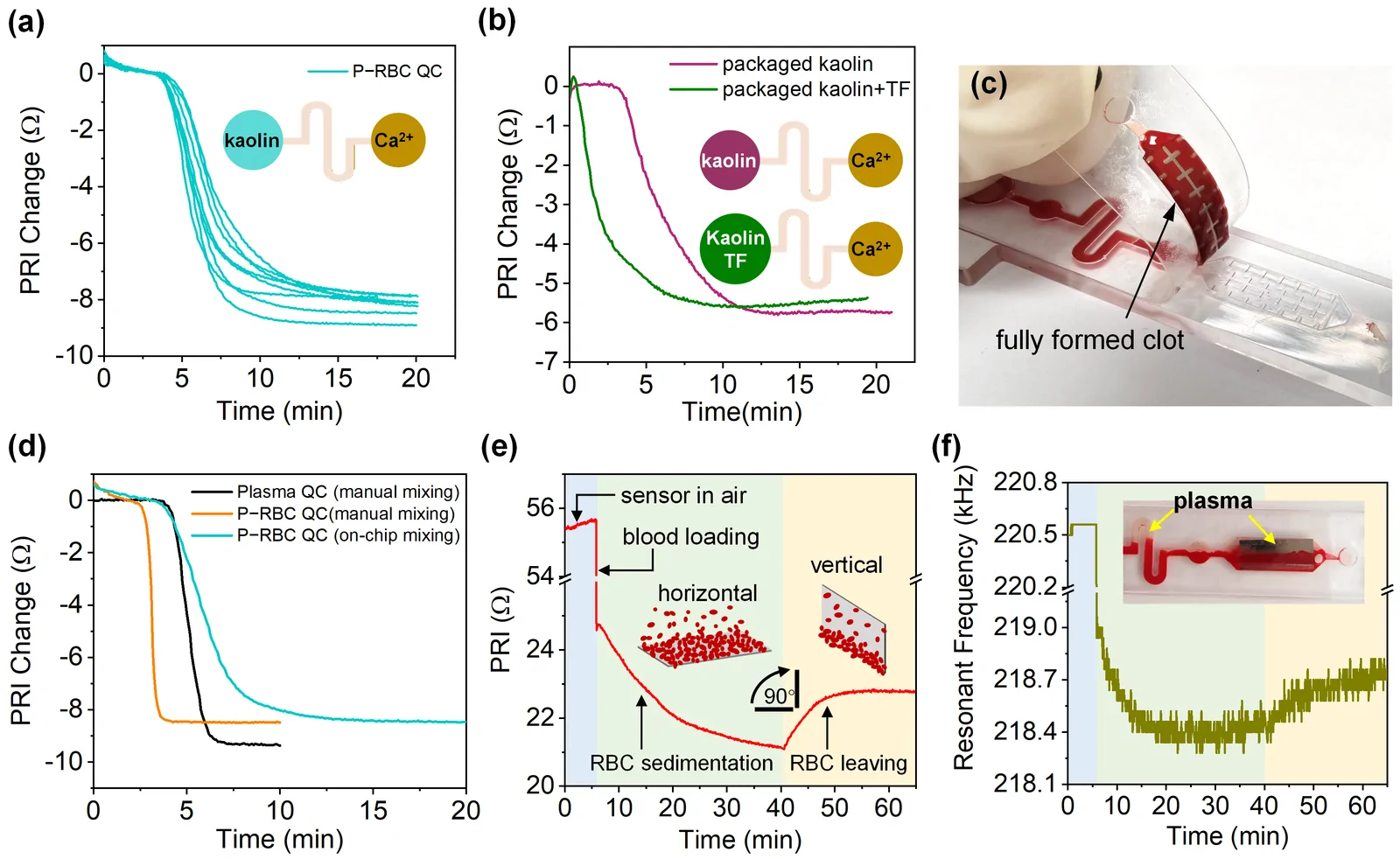
Reproducibility evaluation of the ME sensing chip with prepackaged lyophilized reagents, and the characterization of erythrocyte sedimentation effects. (**a**) Real-time PRI changes from clotting measurements (*n* = 8) of the P-RBC QC samples (inset: layout of prepackaged reagents within the chip). (**b**) Clotting profiles of the clinical WB samples activated by kaolin alone versus a combination of kaolin and tissue factor (TF). Inset: corresponding prepackaged reagents for each condition. (**c**) Photograph of the fully formed blood clot encapsulating the sensor after measurement. (**d**) Comparison of clotting profiles between manual mixing and on-chip passive mixing for kaolin-activated tests. Influence of erythrocyte sedimentation on (**e**) PRI and (**f**) resonant frequency by loading anticoagulated blood onto horizontally and vertically placed chips without reagents. Inset: photograph of plasma separation following the erythrocyte sedimentation.

To demonstrate the reproducibility of the ME sensing chip with a prepackaged kaolin activator for intrinsic activation, eight replicate measurements of the P-RBC QC samples were performed, and the coefficients of variation (CV) for the clotting parameters were evaluated (Figure 5a). Each test reached completion within 20 min. Except for ME-KT, the CV values for ME-RT (5.3%), ME-AG (7.8%), and ME-DA (4.4%) were all well below 10%, successfully meeting typical clinical standards. The mean value of ME-KT was 1.95 min; such a relatively small value can readily lead to a higher CV. These statistical data are summarized in Table S2. The reproducibility errors in coagulation assays originate from variations in chip fabrication, the measurement precision of the equipment, and the dissolution efficiency and passive mixing of the on-chip lyophilized reagents. Nevertheless, the results presented in Figure 5a further confirm that the optimized lyophilized reagent recipe ensures adequate coagulation activation throughout the reaction process, without significant variations. Furthermore, clinical WB samples were measured to verify the biological activity of the prepackaged reagents (Figure 5b). Dual activation via kaolin and TF exhibited an accelerated reaction process compared with activation by kaolin alone, yielding a shorter ME-RT (0.58 min vs. 3.33 min), a shorter ME-KT (0.5 min vs. 0.75 min), and a larger ME-AG (69.7° vs. 53.1°). These results confirm the effective activation of both intrinsic and extrinsic coagulation pathways and the ability to detect coagulation pathway deficiencies. Notably, both clotting tests were completed within 15 min, whereas commercial TEG analyzers require up to 30 min for a single assay, which may be impractical for urgent clinical cases [6,14]. However, the resonant frequency of the ME sensors exhibited negligible shifts (Figure S5), contrasting sharply with the prominent frequency shift (~0.5 kHz) observed in the P-RBC QC samples (Figure S4). This discrepancy may be attributed to the complex components of WB samples, which may exert a mutually inhibitory effect on the mechanical resonance of the ME sensor. Figure 5c shows the fully formed clot completely encapsulating the sensor after measurement. Although on-chip passive mixing does not achieve the homogenous mixing of the sample with the lyophilized reagents within the microchannel, localized coagulation is sufficient to propagate throughout the entire sensing domain. To further elucidate the impact of on-chip passive and active mixing on clotting profiles, a comparative test was performed by manually mixing the sample with liquid kaolin in a centrifuge tube by pipette prior to loading. As shown in Figure 5d, manual mixing accelerated the reaction process, achieving completion within 10 min, whereas on-chip mixing slowed down the process. Despite this difference in reaction efficiency, the on-chip method consistently completed each test within 15 min with good repeatability, highlighting the high quality of the fabricated device.

Interestingly, a slight downward trend in the clotting profiles was observed before clot formation in samples containing RBCs (Figure 5d, orange and blue lines), whereas this phenomenon was entirely absent in plasma-only samples (Figure 5d, black line). We therefore hypothesized that this effect was caused by changes in mass loading on the ME sensor surface induced by erythrocyte sedimentation. To verify this hypothesis, the sensor was positioned either horizontally, allowing its surface to catch settling RBCs, or vertically, with its surface parallel to the direction of RBCs sedimentation. Upon loading anticoagulated WB into a horizontally placed chip without reagents, the PRI of the sensor gradually decreased over 35 min (Figure 5e, green region), accompanied by a notable downward resonant frequency shift due to the mass loading effect (Figure 5f). Rotating the chip by 90° to a vertical orientation resulted in an immediate signal inversion as the sedimented RBCs detached and left the sensor surface (Figure 5e,f, yellow region). The inset in Figure 5f displays the plasma formed via erythrocyte sedimentation following 20 min of vertical placement. These results prove that erythrocyte sedimentation indeed affects the measurement signal before clot formation. Nevertheless, this unique mass loading signature also provides a new insight for utilizing this ME sensing platform to evaluate the erythrocyte sedimentation rate (ESR) simultaneously.

### 3.3. Heparin Assessment of Clinical Samples

**Figure 6:**
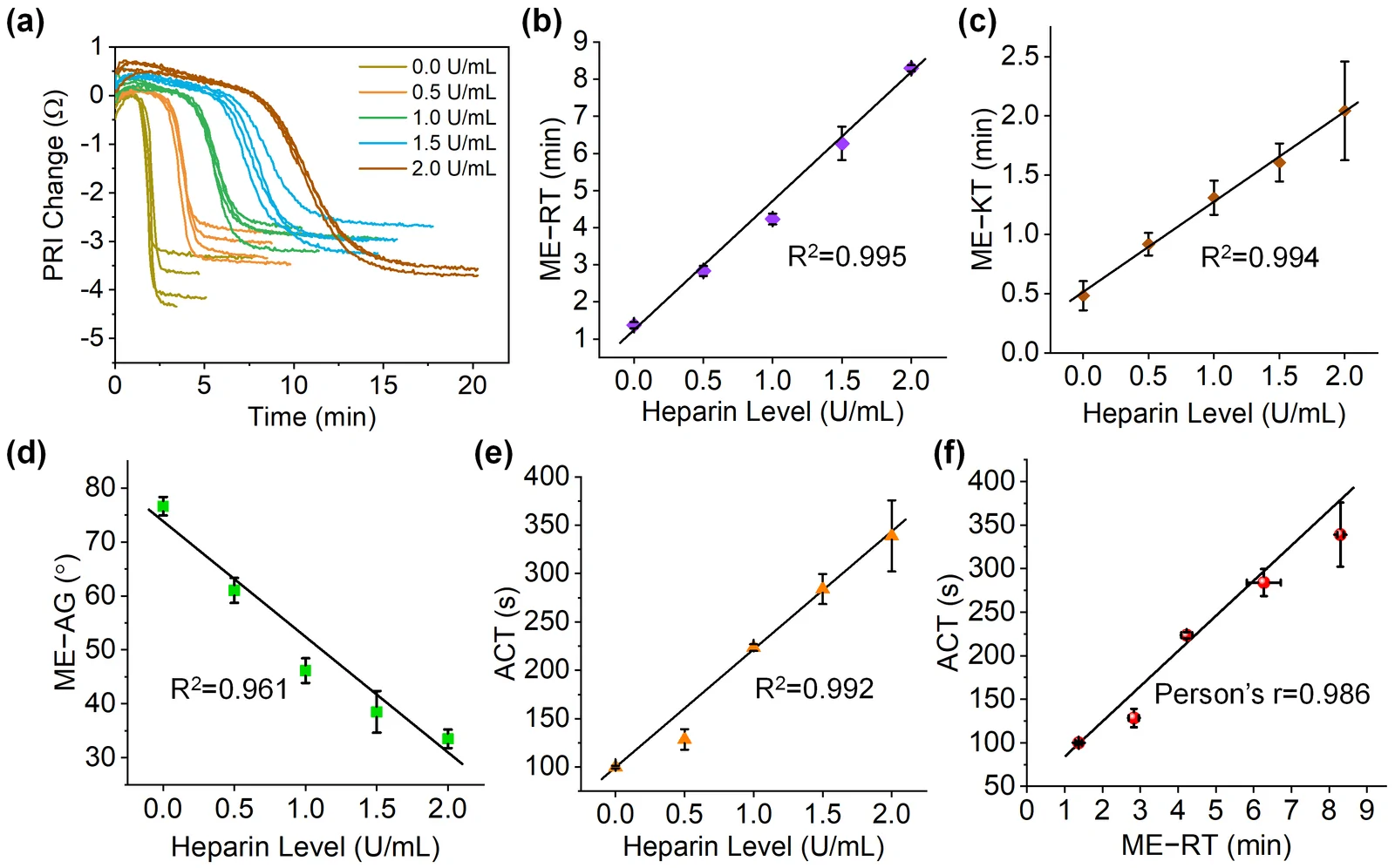
Evaluation of heparin anticoagulation using clinical WB samples. (**a**) Clotting profiles of heparinized blood with varying concentrations (*n* = 4 for per level). Linear regressions of (**b**) ME-RT, (**c**) ME-KT, and (**d**) ME-AG against heparin levels (data points represent mean ± standard deviation (SD) and *n* = 4). (**e**) The measured activated clotting time (ACT) reference results by the commercial ACT Plus^®^ analyzer for the identical heparinized blood samples (data points represent means ± SD and *n* = 2). (**f**) Pearson’s correlation analysis between the clinical ACT values and the ME sensing chip-derived ME-RT.

To further demonstrate the utility of the ME sensing chip for rapid coagulation assessments, heparin anticoagulation monitoring was performed according to the protocol described in Section 2.7. Heparin prolongs clotting time by enhancing the activity of antithrombin III, which inhibits key clotting factors, primarily thrombin (factor IIa) and factor Xa. Near-patient heparin monitoring provides real-time quantitative assessments of anticoagulation state to guide the precise dosing of heparin and its reversal agent, which is critical for balancing the risk of thrombosis against life-threatening bleeding during major surgeries, such as cardiopulmonary bypass. Here, the clotting profiles of heparinized clinical WB samples with a concentration range of 0–2 U/mL were monitored using the ME sensing chips (Figure 6a). The results clearly demonstrate that the coagulation process is correspondingly prolonged as the heparin level increases. Additionally, the erythrocyte sedimentation becomes pronounced before clot formation, especially when the heparin concentration exceeds 1 U/mL and the clotting time extends beyond 5 min. Generally, the exact anticoagulation effect achieved per unit dose (U/mL) of heparin varies widely depending on individual patient hematological characteristics. The ME-RT values derived from these clotting profiles exhibited a good linear correlation (R^2^ = 0.995) with the heparin concentration, and a sensitivity of 3.5 min/unit (208.6 s/unit), as shown in Figure 6b. For the blood sample, the ME-RT exceeded 8 min at a heparin level of 2 U/mL, satisfying the clinical targets for ACT monitoring (typically >480 s during major surgeries) [43]. The parameters ME-KT and ME-AG also exhibited high sensitivity to heparin levels (Figure 6c,d), indicating that high-dose heparin not only delays clot initiation (prolonged ME-RT), but also significantly decelerates the rate of clot formation (i.e., an elevated ME-KT and a reduced ME-AG). Consequently, the clinical interpretation of ME-KT and ME-AG must be integrated with ME-RT to differentiate anticoagulation levels and to recognize abnormalities attributable to non-heparin-related coagulopathies.

Figure 6e shows the comparative ACT results obtained from identical WB samples using the commercial ACT Plus^®^ analyzer, exhibiting a linear relationship (R^2^ = 0.992) with heparin levels and a sensitivity of 122.0 s/unit. At a heparin level of 2 U/mL, the ACT reached 339 s, which is shorter than the corresponding ME-RT. The discrepancy in clotting time can be attributed to the higher temperature (37 °C) of the commercial analyzer, which thermally accelerates the enzymatic coagulation cascade; however, this temperature difference does not change the underlying cascade process. Furthermore, Pearson’s r correlation analysis revealed an excellent correlation coefficient of r = 0.986 between the two methods (Figure 6f), validating the accuracy of the developed ME sensing chip and highlighting its potential for use in bedside monitoring. ME-RT exhibits a higher sensitivity compared with clinical ACT and can serve as a functional equivalent to the endpoint-based clinical ACT. The accompanying ME-KT and ME-AG provide supplementary insights into clot growth kinetics, offering clinicians comprehensive information for bleeding risk assessment. Moreover, the blood consumption for the ME sensing chip is only 46 μL, which is one order of magnitude lower than that of the ACT Plus^®^ analyzer (400 μL), making the platform highly patient-friendly, particularly for patients suffering severe blood loss or disseminated intravascular coagulation (DIC). According to public procurement prices from hospitals in China, a commercial Medtronic HR ACT cartridge costs approximately 7.80 USD per test, which highlights the material cost advantage of our developed chip. Meanwhile, benefiting from the pumpless design without external transmission components, future developments in ME sensing-based devices will make them smaller and more portable than current commercial devices.

### 3.4. Sensitivity of Clotting Parameters to Blood Components

**Figure 7:**
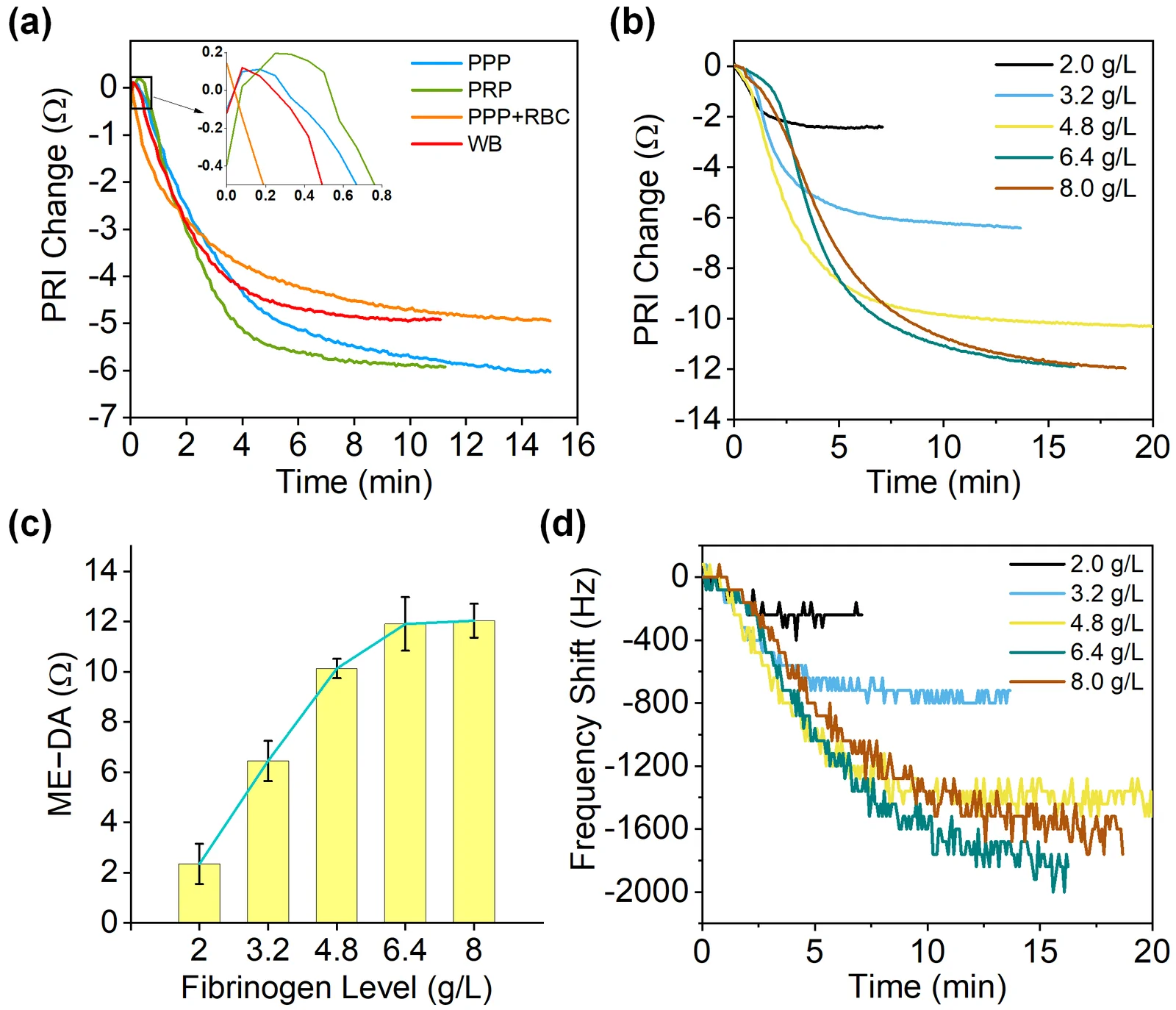
Specific sensitivity of clotting parameters to cellular and molecular blood components. (**a**) Clotting profiles derived from platelet-poor plasma (PPP), platelet-rich plasma (PRP), PPP+RBC, and WB samples (inset: enlarged view highlighting localized curves). (**b**) Clotting profiles recording the fibrin mesh generated via the thrombin cleavage of varying fibrinogen concentrations. (**c**) Corresponding ME-DA values quantifying the final clot strength of the resultant fibrin mesh (data points represent means ± SD and *n* = 3). (**d**) Real-time changes in resonant frequency during fibrin clot formation.

Coagulation is a highly coordinated, multi-stage process driven by the concerted actions of coagulation factors, fibrinogen, and platelets. The clotting parameter ME-RT directly reflects coagulation factor activity, and has been validated across the intrinsic pathway (activated by kaolin via factor XII), the extrinsic pathway (activated by TF via factor VII), and heparin-induced anticoagulation (which inhibits factors Xa and IIa). Furthermore, the coagulation kinetics indices, ME-KT and ME-AG, which track the clot formation rate have demonstrated high sensitivity to the level of heparinization. Previous studies have established that total clot strength is co-determined by platelets’ activity and fibrin formation, wherein platelets generate clot retraction stress, and the fibrin mesh provides the foundational elastic modulus [44,45]. To systematically analyze the relationship between the sensor’s response parameter ME-DA and specific clot components, comparative tests were performed using PPP, PRP, PPP+RBC, and WB samples (Figure 7a). The ME-DA values for PPP and PRP were nearly identical, and similar parity was observed between PPP+RBC and WB. This finding suggests that the ME sensor is selectively insensitive to platelet-mediated viscoelasticity. Additionally, RBC-containing samples (PPP+RBC and WB) exhibited lower ME-DA values compared with RBC-free plasma samples (PPP and PRP). This can be attributed to the physical embedding of trapped erythrocytes, which disrupts the continuity of the fibrin network and structurally weakens the clot elasticity [46].

To further validate the ME sensor’s sensitivity toward fibrin clotting, we measured fibrin gelation using bovine fibrinogen (final concentrations ranging from 2.0 to 8.0 g/L) cleaved by thrombin (500 U/mL) at a 4:1 volume ratio. Because the final amount of the formed fibrin is primarily governed by the fibrinogen concentration, it was accurately quantified by ME-DA in a positively correlated manner (Figure 7b,c). The linear range of MA-DA for fibrinogen levels spans from 2.0 to 4.8 g/L. Given that the normal range of human fibrinogen is 2.0–4.0 g/L, the detection window is fully capable of identifying abnormal fibrinogen function. Consequently, the proposed ME sensing chip offers a distinct advantage in clinical settings where monitoring functional fibrinogen status is the primary therapeutic priority, such as in liver dysfunction [47,48], cardiovascular diseases [49], trauma-induced coagulopathy [50], and thrombo-inflammatory therapies [51]. Notably, the resonant frequency tracked at the PRI exhibits a marked response to fibrin clotting, yielding downshifts of −240 Hz at 2.0 g/L and −1390 Hz at 4.8 g/L (Figure 7d). According to the principle that resonant frequency is highly sensitive to liquid viscosity [39], these results imply that the fibrin network formed with increasing fibrinogen levels can increase not only the elasticity of the clot but also its viscosity. This pronounced downward frequency shift contrasts sharply with the small upward shift observed during the coagulation of the P-RBC QC (Figure S4) and the negligible change observed for clinical WB samples (Figure S5). This phenomenon strongly suggests that molecular and cellular blood components exert competing, opposing mechanical forces on frequency responses. Consequently, a dual-mode approach (resonant frequency and impedance amplitude) enables the comprehensive evaluation of clot viscosity and elasticity for non-whole-blood matrices. For complex WB samples, the resonant frequency mode is difficult to utilize, rendering the amplitude-based mode a more robust metric for characterizing coagulation.

These results confirm the utility of the ME sensor for the rapid assessing of coagulation factors and fibrin-related disorders, despite its limitation in evaluating platelet function. Benefiting from pumpless operation, low sample consumption, and prepackaged reagents, the ME sensing chip holds great promise for a decentralized POC testing platform. Given these advantages, this platform is highly applicable to clinical emergencies like major surgeries, postpartum hemorrhage, and trauma triage, as well as home monitoring for hemophilia and oral anticoagulant therapies. Future work will focus on optimizing the on-chip passive mixing strategy to enhance reproducibility and conducting a deeper physical interpretation of the clotting parameters across different clot staging behavior. Meanwhile, a portable device prototype is currently being developed to facilitate clinical validation and trials in future applications.

## 4. Conclusions

In summary, we have developed an ME sensor-based pumpless microfluidic chip for rapid, user-friendly coagulation assessment. The platform requires a blood volume of only 46 μL, delivers results within 15 min, and features prepackaged lyophilized reagents, making it well-suited for POC applications. The estimated fabrication cost is 1.66 USD per chip, which underscores its high commercial viability for disposable use. Replicate tests using P-RBC QC samples confirmed robust reproducibility, with coefficients of variation below 10% for clotting parameters (ME-RT, ME-AG, ME-DA). The on-chip coagulation activation of both extrinsic and intrinsic pathways was successfully demonstrated using clinical samples. Furthermore, the heparin monitoring results correlated well with the clinical standard, highlighting the platform’s potential as a practical, decentralized alternative to conventional ACT testing. Sensitivity evaluation toward specific blood components further validated the ME sensor’s dual-mode characterization capability, while also confirming its limitation in terms of insensitivity to platelet function. Further work will include optimizing on-chip mixing efficiency and developing a portable device to support clinical use.

## Data Availability

The data presented in this article are available on request from the corresponding author.

## Supplementary Materials

The following supporting information can be downloaded at: https://www.mdpi.com/article/10.3390/bios16080429/s1, Figure S1. Schematic of the experimental measuring setup, including detection module of the ME sensing chip, impedance analyzer, and a PC with LabVIEW program (Enlarged view: detail of the detection module); Figure S2. Simulation modelling of blood flow through the ME sensing chip under laminar flow field. (a) Calculated flow streamlines within the entire computational domain at an inlet velocity of 5 mm/s. (b) Simulated shear rate across the entire domain at an inlet flow velocity of 5 mm/s. The maximum wall shear rate (approximately 150 s^−1^) appears at the microchannel corners (see magnified view). Labels A, B, C, D, E, and F denote the wall of micropillars within micropump. (c) Flow velocities distribution within the micropump. Inset: flow velocity map at the internal cross section. The x-axis represents the position of internal cross section. (d) Simulated wall shear rates at the wall of micropump (labeled A, B, C, D, E, and F). (e) Inner shear rates between adjacent micropillars within micropump; Figure S3. The dissolving process of red-dyed lyophilized CaCl_2_ at different time in glycerol-water solution (viscosity: 5.4 mPa·s) within the sensing chip. The last image shows the back side of the chip; Figure S4. Real-time monitoring of resonant frequency during coagulation process of the P-RBC QC sample using the ME sensing chip with prepackaged activators; Figure S5. Real-time monitoring of resonant frequency during coagulation process of clinical whole blood (WB) samples using the ME sensing chip with prepackaged activators (kaolin alone versus kaolin combined with tissue factor (TF)); Table S1. Detailed cost breakdown per ME sensing chip; Table S2. Statistical data of replicate coagulation measurements of P-RBC QC samples using the ME sensing chip. The chip was packaged with kaolin and CaCl_2_. The listed clotting parameters were derived from clotting curves in Figure 5a. Reference [52] is cited in the Supplementary Materials.
