# Supplementary material for: Magnetoelastic Sensors-Based Pumpless Microfluidic Chip for Point-of-Care Coagulation Kinetics Monitoring
Source: Biosensors (Basel). 2026 Aug 6;16(8):429. doi: 10.3390/bios16080429 (PMC13511441; doi:10.3390/bios16080429)
Supplement: Supplementary file 1 [file biosensors-16-00429-s001.zip › biosensors-4437999-supplementary.pdf]

biosensors

Supplementary

Magnetoelastic Sensors-Based Pumpless Microfluidic Chip for Point-of-Care Coagulation Kinetics Monitoring

Yao Lu [1,2], Weiguo Liang [1,2], Jun Qian [1,2], Junpo Li [1], Shengpeng Wu [1], Mao Xia [3] and Haixuan Sun [1,2,*]

[1] Suzhou Institute of Biomedical Engineering and Technology, Chinese Academy of Sciences, Suzhou 215163, China; luyao@sibet.ac.cn (Y.L.); liangwg@sibet.ac.cn (W.L.); qianj@sibet.ac.cn (J.Q.); lijp@sibet.ac.cn (J.L.); wusp@sibet.ac.cn (S.W.)
[2] School of Biomedical Engineering (Suzhou), University of Science and Technology of China, Hefei 230026, China
[3] Affiliated Hospital of Nantong University, Nantong 226001, China; xiamao0126@163.com
* Correspondence: sunhx@sibet.ac.cn

Experimental Setup

Figure S1 illustrates the experimental setup for the magnetoelastic (ME) sensing chip. The sensing chip was inserted into a pick-up coil (73 turns, 32 AWG wire, diameter 15 mm), with the longitudinal directions of both components oriented parallel to each other (see the magnified exploded view for details). In the experiment, a permanent magnet was placed at a distance from the ME sensor to provide a static bias magnetic field of 17 Oe. An alternating current (~5 mA) was applied to the pick-up coil by an impedance analyzer (Micro Test 6632, SAIMR), thereby exciting resonant vibration in the ME sensor via the magnetostrictive effect. The magnetization state of the ME sensor could be wirelessly detected by the pick-up coil through electromagnetic induction. Accordingly, the electrical signal, represented by the impedance–frequency curve, was measured by the impedance analyzer. In this work, the frequency sweep was centered at the resonant frequency (~220 kHz), with a scanning range of 10 kHz and a frequency resolution of 40 Hz. The characteristic peak of the impedance–frequency curves, which were sensitive to the loading properties, were monitored in real time using a custom-built LabVIEW program, with a sampling interval of 5 seconds.

Blood Flow Simulation

A 3-dimensional laminar field model was established using COMSOL 6.0 software to investigate the shear effect exerted on blood in the microchannel of the chip. In this model, a Carreau fluid is used to capture the non-Newtonian behavior of blood. The apparent viscosity, denoted as μ_{app}, of a Carreau fluid is defined as [1]

$$\mu_{app} = \mu_{inf} + \left(\mu_0 - \mu_{inf}\right)[1 + \lambda\dot{\gamma}^2]^{\frac{n-1}{2}}$$

where the μ_{inf}=0.00345 Pa·s, μ_0=0.056 Pa·s, λ=3.313 s, $\dot{\gamma}$, and n=0.3568 are viscosity at infinite shear rate, viscosity at zero shear rate, the relaxation time, the shear rate and the power index, respectively. A no-slip boundary condition was applied in the laminar flow field. Consistent with the capillary force-driven flow observed in the ME sensing chip, inlet velocities were set in a range of 3-7 mm/s, and a zero pressure was applied at the outlet boundary condition. A mesh consisting of 3×10^5 domain elements yielded the optimal resolution for the flow simulations.

The simulation velocity for blood at an inlet flow velocity of 5 mm/s is shown in Figure S2(a-b). The blood flow velocity reached approximately 9.5 mm/s in the micro-

Received: 30 June 2026
Revised: 27 July 2026
Accepted: 3 August 2026
Published: 6 August 2026

Copyright: © 2026 by the authors. Submitted for possible open access publication under the terms and conditions of the Creative Commons Attribution (CC BY) license.

channel and 5.4 mm/s in the micropump region. The shear rates for blood across the entire device are shown in Figure S2(c), with the maximum shear rate (~150 s^{-1}) appearing at the microchannel corners. The wall shear rates for blood exerted at the micropump (labeled as A, B, C, D, E, and F) ranged from 10 to 100 s^{-1} (Figure S2(d)). Furthermore, the gap between adjacent micropillars in the micropump was 1.25 mm, and the inner shear rates experienced by blood flowing through this region were ~10 s^{-1}, which were considerably lower than the wall shear rates (Figure S2e). These simulation results confirm that no shear-induced damage to blood would occur within the ME sensing chip.

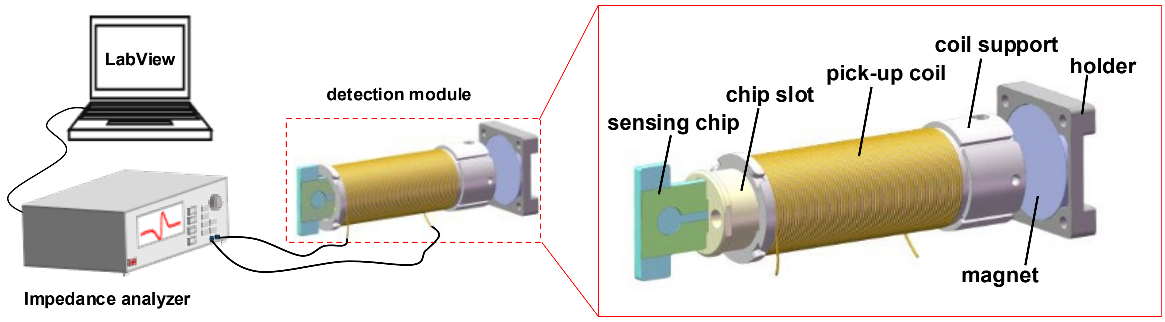

Figure S1. Schematic of the experimental measuring setup, including detection module of the ME sensing chip, impedance analyzer, and a PC with LabVIEW program (Enlarged view: detail of the detection module).

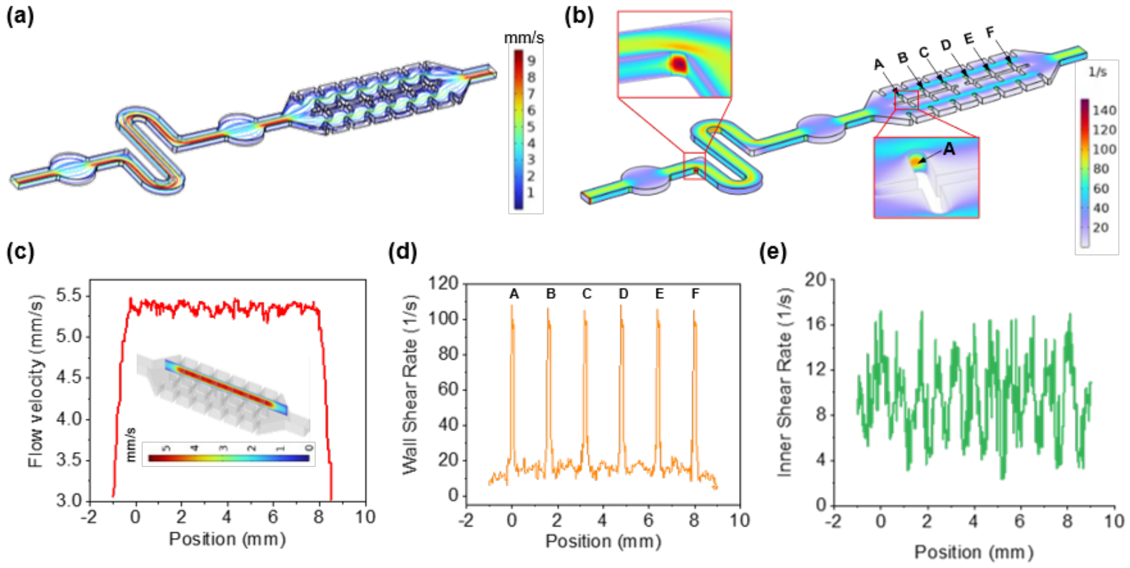

Figure S2. Simulation modeling of blood flow through the ME sensing chip under laminar flow field. (a) Calculated flow streamlines within the entire computational domain at an inlet velocity of 5 mm/s. (b) Simulated shear rate across the entire domain at an inlet flow velocity of 5 mm/s. The maximum wall shear rate (approximately 150 s^{-1}) appears at the microchannel corners (see magnified view). Labels A, B, C, D, E, and F denote the wall of micropillars within the micropump. (c) Flow velocities' distribution within the micropump. Inset: flow velocity map at the internal cross section. The x-axis represents the position of the internal cross section. (d) Simulated wall shear rates at the wall of micropump (labeled A, B, C, D, E, and F). (e) Inner shear rates between adjacent micropillars within the micropump.

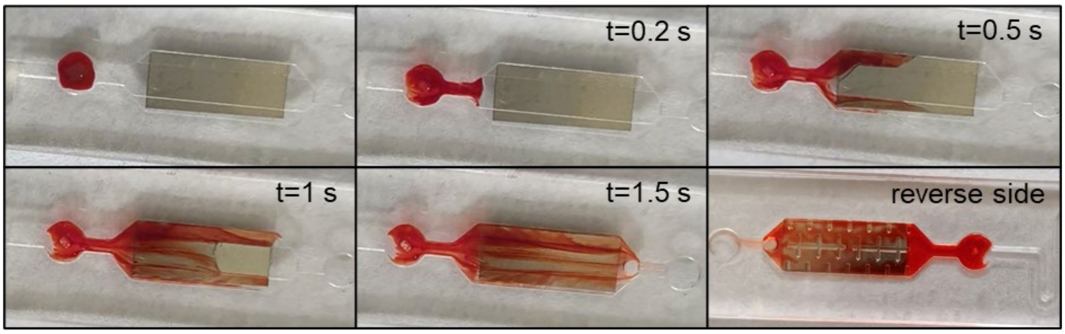

Figure S3. The dissolving process of red-dyed lyophilized $CaCl_2$ at different times in the glycerol–water solution (viscosity: 5.4 mPa·s) within the sensing chip. The last image shows the back side of the chip.

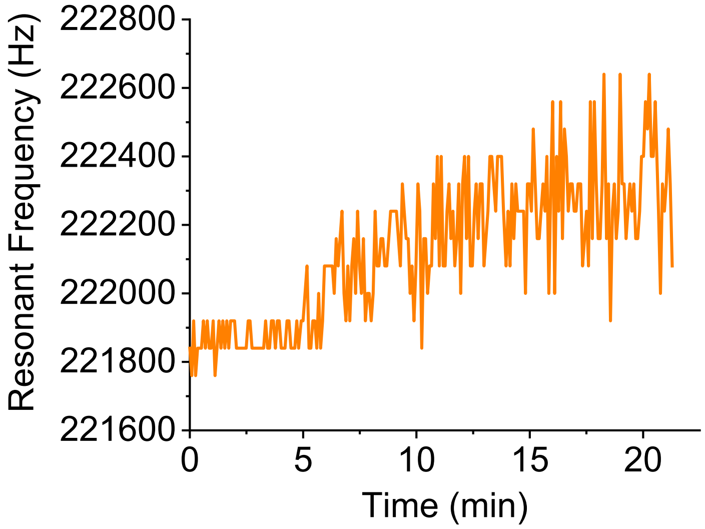

Figure S4. Real-time monitoring of resonant frequency during coagulation process of the P-RBC QC sample using the ME sensing chip with prepackaged activators.

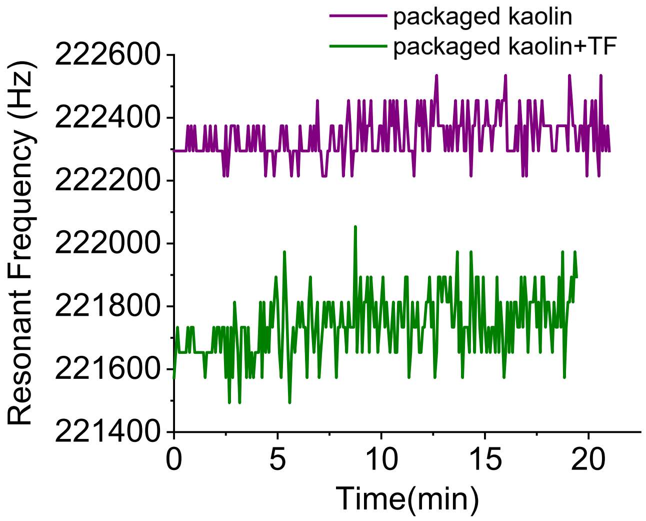

Figure S5. Real-time monitoring of the resonant frequency during the coagulation process of clinical whole blood (WB) samples using the ME sensing chip with prepackaged activators (kaolin alone versus kaolin combined with tissue factor (TF)).

Table S1. Detailed cost breakdown per ME sensing chip.

Item	Small-batch pilot (<2000)
**amorphous alloy ribbon**	$0.36/m ($0.0036/sensor)
**laser cutting**	$0.59
**injection**	$0.72
**die-cutting (including material)**	$0.30
**hydrophilic reagent**	$0.01
**on-chip reagents**	$0.03
**total**	$1.6536

Note: All costs listed above are material and processing costs only. Packing, utilities (water/electricity), and labor are not included.

Table S2. Statistical data of replicate coagulation measurements of P-RBC QC samples using the ME sensing chip. The chip was packaged with kaolin and $CaCl_2$. The listed clotting parameters were derived from clotting curves in Figure 5(a).

	ME-RT	ME-KT	ME-AG	ME-DA
**1**	3.67	1.75	61.88	8.07
**2**	3.67	1.58	68.17	7.90
**3**	4.17	2.00	54.69	8.25
**4**	3.83	1.75	61.69	7.86
**5**	4.00	2.33	55.53	8.08
**6**	4.00	2.42	58.49	7.86
**7**	3.75	1.75	66.26	8.89
**8**	3.58	2.00	62.00	8.47
**Mean**	3.83	1.95	61.09	8.17
**STD**	0.204	0.299	4.472	0.359
**CV**	5.34%	15.37%	7.76%	4.40%

Reference

1. He, F.; Wang, X.; Hua, L.; Guo, T. Non-Newtonian Effects of Blood Flow on Hemodynamics in Pulmonary Stenosis: Numerical Simulation. Appl. Bionics Biomech. **2023**, 1434832. https://doi.org/10.1155/2023/1434832.

https://doi.org/10.3390/bios16080429
